# Exploring the association of interleukin polymorphisms with aggression and internalizing behaviors in children and adolescents

**DOI:** 10.1002/brb3.2753

**Published:** 2022-09-28

**Authors:** Jennie G. Pouget, Lyubov Bryushkova, Emiko Koyama, Clement C. Zai, Trehani M. Fonseka, Daniel Mueller, James L. Kennedy, Joseph H. Beitchman

**Affiliations:** ^1^ Campbell Family Mental Health Research Institute Centre for Addiction and Mental Health Toronto Canada; ^2^ Department of Psychiatry University of Toronto Toronto Canada

**Keywords:** adversity, aggression, child, cytokine, gene, inflammation, interleukin, internalizing, trauma

## Abstract

**Background:**

There is growing evidence that inflammation influences mental health. Blood interleukin levels, which regulate inflammation, have been linked to aggression and internalizing behaviors. We performed a hypothesis‐driven genetic study to (1) evaluate the association of *IL1B*, *IL2*, and *IL6* gene variants with aggression and internalizing behaviors and (2) explore gene–environment interactions with childhood adversity in a deeply phenotyped childhood‐onset aggression sample including 255 cases and 226 controls of European ancestry.

**Methods:**

We evaluated the association of putative functional and tag SNPs within *IL1B*, *IL2*, and *IL6* with aggression case status, parent‐reported internalizing problems, self‐reported anxiety symptoms, and self‐reported depressive symptoms in our sample. We also performed exploratory GxE analyses within cases, testing for statistical interaction between interleukin SNP genotype and childhood adversity for depressive symptoms.

**Results:**

No significant association was observed between any of the interleukin SNPs and childhood‐onset aggression. We observed association of *IL6* variant rs2069827 with depressive symptoms (*p* = 7.15×10^–4^), and trends for an interaction between severe childhood adversity and SNPs in *IL1B* and *IL2* for depressive symptoms.

**Conclusions:**

Our findings provide preliminary evidence that common variation in *IL6* may be associated with depressive symptoms in children and adolescents, and that common variation in interleukin genes may sensitize individuals to the depressogenic effects of traumatic life experiences. Replication in independent samples is needed.

## Graphical abstract

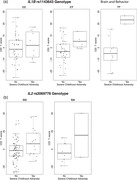

## 1 INTRODUCTION

Behavioral problems in young children generally fall into two broad categories: externalizing behaviors (problems with attention, self‐regulation, and noncompliance including inattention/hyperactivity, antisocial, and aggressive behaviors) and internalizing behaviors (depression, withdrawal, anxiety, and somatic complaints) (Achenbach et al., 1987; Campbell, 1995). Both externalizing and internalizing behaviors in childhood and adolescence are associated with long‐term difficulties, including poor functional and mental health outcomes (Achenbach et al., 1995; Campbell and Ewing, 1990; Hughes et al., 2008; Johnson et al., 2018; Moffitt, 1990; Pine et al., 1998). Among children, aggression and internalizing behaviors (including depression and anxiety) are the most common reasons for referral to psychiatric care (Pikard et al., 2018), and we have therefore focused our attention on these clinical entities in the present study while recognizing that aggression alone does not represent the full spectrum of externalizing behaviors.

Currently, the causes of childhood‐onset aggression and internalizing disorders are not fully understood, and treatment options remain limited. While certain psychotherapy modalities (e.g., parent management training (Michelson et al., 2013) and cognitive‐behavioral therapy (Sukhodolsky et al., 2000)) have been shown to improve aggressive and internalizing disorders in children, they are not widely available. As a result, second‐generation antipsychotics (SGAs) and selective serotonin +/‐ norepinephrine reuptake inhibitors (SSRIs and SNRIs) are often used in children and adolescents to treat severe aggression and depression/anxiety, respectively, despite limited evidence of efficacy (Locher et al., 2017; Pringsheim and Gorman, 2012) and significant side‐effect burden (Locher et al., 2017; Pringsheim et al., 2011) particularly for SGAs. An improved understanding of the causes of childhood‐onset aggression and internalizing behaviors is needed to facilitate improvements in prevention, detection, and management. Notably, both aggression and internalizing behaviors are heritable (*h* ^2^ ∼ 0.5 (Bergen et al., 2007; Rhee and Waldman, 2002)) making them amenable to genetic study.

There is accumulating support for an “inflammatory hypothesis” of aggression and internalizing behaviors, whereby some complex interplay of biological, psychological, and social risk factors triggers an overactive inflammatory response, which then predisposes an individual to develop aggression, depression, and anxiety (Costello et al., 2019; Miller and Raison, 2016; Takahashi et al., 2018). Initial support for this inflammatory hypothesis came from the observation in cross‐sectional studies that peripheral inflammation was present among patients with certain medical and psychiatric disorders—including aggression and internalizing behaviors (Costello et al., 2019; Miller and Raison, 2016; Takahashi et al., 2018). Longitudinal studies have also found that baseline blood levels of inflammatory biomarkers—including C‐reactive protein (CRP), interleukin 1 beta (IL1B), and interleukin 6 (IL6)–predict the development of both aggressive and internalizing behaviors in animal and human studies (Costello et al., 2019; Graham et al., 2006; Lamers et al., 2019; Valkanova et al., 2013). Furthermore, there is a growing body of literature identifying elevated peripheral inflammatory biomarkers, such as CRP and IL6, in children and adolescents with externalizing (Mitchell and Goldstein, 2014; Pham et al., 2022; Slopen et al., 2013) and internalizing behaviors (Belem da Silva et al., 2017; Copeland et al., 2012; Mitchell and Goldstein, 2014; Pham et al., 2022; Slopen et al., 2013). A recent prospective study of 482 infants found that higher glycoprotein acetyls (GlycA, a measure of cumulative inflammation) levels at birth were associated with externalizing and internalizing problems at age two, underscoring the impact of inflammation during sensitive periods of early brain development (Pham et al., 2022). Overall, it appears that inflammation may be an important biological pathway underlying aggressive and internalizing behaviors in at least a subset of children and adolescents. For these young people, various combinations of environmental exposures and genetic vulnerability lead to peripheral inflammation, which ultimately may contribute to their psychiatric symptoms.

In terms of relevant environmental exposures predisposing to early life aggression and internalizing behaviors, early life stress is a well‐established risk factor (Dodge et al., 1990; Nemeroff, 2016). Childhood neglect and physical/emotional/sexual abuse influence mental health throughout life, including increasing severity of both externalizing and internalizing symptoms (Anda et al., 2006; Cole et al., 2012; Janusek et al., 2017; Tursich et al., 2014). While the neurobiological mechanisms by which childhood adversity influences mental health are not fully understood, it is clear that early‐life stress has cumulative and lasting effects on both the brain (Anda et al., 2006) and the immune system (Cole et al., 2012; Saarinen et al., 2021). An emerging theory is that childhood adversity increases cross‐talk between the immune system and the brain throughout the lifespan, resulting in sensitization of the brain's threat responses (cortico‐amygdala circuit) and attenuation of reward responses (cortico‐basal circuit) and executive control (prefrontal cortex) via pro‐inflammatory cytokines—including IL1B and IL6—acting on these brain regions (Nusslock and Miller, 2016). Thus, childhood adversity appears to influence the development of aggressive and internalizing behaviors, at least in part, by altering levels of circulating inflammatory biomarkers that influence the developing brain.

Regarding genetic risk factors, large‐scale genome‐wide association studies (GWASs) of childhood aggression (Pappa et al., 2016) and internalizing behaviors (Benke et al., 2014; Jami et al., 2020; Trzaskowski et al., 2013) have revealed that these traits are polygenic and share genetic risk factors with adult‐onset disorders, but have not yet identified robustly associated genetic variants. To increase statistical power for variant discovery in these GWASs, the results of many smaller studies were pooled by meta‐analysis in order to achieve massive sample sizes (*N*_range_ = 2,000–60,000). Trade‐offs for increasing statistical power by pooling smaller studies include limited phenotypic data and increased clinical heterogeneity. Hypothesis‐driven studies are an alternative strategy to GWASs, which can be used to explore biological hypotheses that require more detailed phenotypic data, such as gene–environment interactions (Caspi et al., 2002). Due to the smaller sizes typical of deeply phenotyped samples, hypothesis‐driven genetic studies are susceptible to false negatives (type II error), as well as spurious findings (type I error) unless there is stringent correction for multiple testing, and are therefore exploratory until robust replication in independent studies has been achieved (Dick et al., 2015). Given the demonstrated role of IL1B, interleukin 2 (IL2), and IL6 in stress‐induced aggression and internalizing behaviors in feline (Bhatt et al., 2005; Hassanain et al., 2003) and rodent models (Chourbaji et al., 2006; Koo and Duman, 2008; Niraula et al., 2019), respectively, polymorphisms in these genes are of particular interest for follow‐up in hypothesis‐driven gene–environment studies.

To date, there has been limited investigation of the relationship between childhood adversity, genetic variation, and aggressive or internalizing behaviors. Previous studies have identified potential interactions between childhood adversity and polymorphisms in *IL1B* and *IL6* in regulating risk for internalizing symptoms (Kovacs et al., 2016; Ridout et al., 2014). Here we performed a hypothesis‐driven genetic study to evaluate the association of *IL1B*, *IL2*, and *IL6* gene variants with aggression and internalizing behaviors in children and adolescents, and to explore gene–environment interactions with childhood adversity.

## 2 MATERIALS AND METHODS

### 2.1 Clinical sample

Our sample comprised 314 cases and 287 controls recruited in an ongoing study of childhood‐onset aggression that has been previously described (Beitchman et al., 2004; Beitchman et al., 2006; Malik et al., 2014). Briefly, cases comprised children and adolescents aged 6–16 years referred to outpatient psychiatric care for persistent, extreme aggression at the Centre for Addiction and Mental Health (a tertiary care hospital providing inpatient and outpatient psychiatric services located in Toronto, Ontario, Canada) and Youthdale Treatment Centre (a community‐based mental health agency providing inpatient and outpatient services in Toronto, Ontario, Canada). Inclusion criteria for cases consisted of a minimum 2‐year history of aggressive behavior, and scores at the 90th percentile or higher on the aggressive subscale of the Achenbach Child Behaviour Checklist (CBCL) by two independent raters, which could include any of: the parent or guardian (CBCL); the teacher (teacher report form, TRF); the participant (youth self‐report form, YSR) (Achenbach and Rescorla, 2001). Healthy controls comprised children and adolescents aged 6–16 years scoring within the normal range on the Achenbach CBCL by two independent raters (Achenbach and Rescorla, 2001) with no history of disruptive behavior, recruited from the community at the Ontario Science Centre (Toronto, Ontario, Canada) and Science North Education Centre (Sudbury, Ontario, Canada). Exclusion criteria for cases and controls included intelligence quotient below 70 as measured using a 2‐subtest shortened form of the Wechsler Intelligence Scale for Children—Third Edition (Weschler, 1991), history of chronic medical illness, psychosis, mania, neurological disorder, or pervasive developmental disorder. Ethnicity was assessed by parental self‐report, and we included only European subjects in our analyses to avoid potential confounding by population stratification.

In addition to case status, information on the following clinical characteristics were analyzed:

*Internalizing problems*. Severity of overall internalizing problems was assessed by parent or guardian report using the Achenbach CBCL internalizing problems score (Achenbach and Rescorla, 2001). The internalizing problems score captures the anxious‐depressed, withdrawn‐depressed, and somatic complaints syndrome scales of the CBCL. Internalizing problems scores were available for 253 cases and 218 controls.

*Anxiety*. Severity of anxiety was assessed by self‐report using Revised Children's Manifest Anxiety Scale (RCMAS) total scores (Reynolds and Richmond, 1985). RCMAS total scores were available for 241 cases and 226 controls.

*Depression*. Severity of depressive symptoms was assessed by self‐report using Children's Depression Inventory (CDI) total scores (Kovacs, 1985). CDI total scores were available for 234 cases and 224 controls.

*Exposure to severe childhood adversity*. Subjects aged 12 and older completed the Massachusetts Youth Screening Instrument—Version 2 (MAYSI‐2), and we assessed history of trauma using the self‐reported MAYSI‐2 Traumatic Experiences scale (Grisso and Barnum, 2000). This scale indicates the number of traumatic experiences endorsed using the following questions: (1) Have you ever in your whole life had something very bad or terrifying happen to you? (2) Have you ever been badly hurt, or been in danger of getting badly hurt or killed? (3) Have you ever been raped, or been in danger of being raped? (4) Have you had a lot of bad thoughts or dreams about a bad or scary event that happened to you? (5) Have you ever seen someone severely injured or killed (in person–not in movies or on TV)? Traumatic life experience data were available for 124 cases and 83 controls. Based on the distribution of scores in our sample (Table 1), we defined severe childhood adversity as a MAYSI‐2 Traumatic Experiences scale score ≥4.

**TABLE 1:** Demographic and clinical characteristics of the sample

Characteristic	Cases (*n* = 255)	Controls (*n* = 226)	*p*
Male (%)	152 (60)	125 (55)	0.39 ^a^
Age at recruitment ± SD (years)	12.06 ± 3.0	11.25 ± 2.45	5.06×10^−5^ ^b^
IQ^c^	97.94 ± 14.0	113.52 ± 14.05	1.79×10^−10^ ^d^
CBCL^e^ total	74.06 ± 6.7	48.31 ± 10.7	5.52×10^−119^ ^d^
CBCL externalizing	74.89 ± 7.5	46.86 ± 9.8	1.78×10^−72^ ^b^
CBCL internalizing	68.66 ± 9.7	49.80 ± 10.2	2.02×10^−52^ ^b^
CDI total^f^	54.73 ± 13.5	43.25 ± 6.6	8.45×10^−28^ ^b^
RCMAS total^g^	52.96 ± 12.4	42.35 ± 10.4	1.55×10^−21^ ^d^
History of abuse (%)^h^	138 (69)	39 (47)	9.92×10^−4^ ^a^

^a^ chi‐square test.

^b^ Kruskal–Wallis test.

^c^ Intelligence quotient (IQ) measured using a 2‐subtest shortened form of the Wechsler Intelligence Scale for Children—Third Edition was available for 236 cases and 42 controls.

^d^ ANOVA test.

^e^ Problem behaviors measured using the Achenbach Child Behaviour Checklist (CBCL) completed by child's parent(s) or guardian were available for 253 cases and 218 controls.

^f^ Self‐reported depressive symptoms measured using the Children's Depression Inventory (CDI) were available for 234 cases and 224 controls.

^g^ Anxiety symptoms measured using the Revised Children's Manifest Anxiety Scale (RCMAS) were available for 241 cases and 226 controls.

^h^ Defined as history of childhood abuse based on physician assessment, which was available for 201 cases and 83 controls.

### 2.2 Genotyping and quality control

#### 2.2.1 Discovery sample

Tag single‐nucleotide polymorphisms (SNPs) with minor allele frequency (MAF) ≥0.05 within 10 kb upstream and 3 kb downstream of each interleukin gene were identified using the database of common SNPs available in the International HapMap Project's Phase III European (CEPH) sample (International HapMap 3 Consortium, 2010) and Haploview version 4.2 (Barrett et al., 2005). SNPs were prioritized for selection based on evidence of regulatory function in the Encyclopedia of DNA Elements (Dunham et al., 2012) as queried using HaploReg (Ward and Kellis, 2012) and RegulomeDB (Boyle et al., 2012). In total, 16 putative functional and tag SNPs within *IL1B* (rs4849127, rs13032029, rs16944, rs3136558, rs1143634, rs1143643), *IL2* (rs2069762, rs2069772, rs2069778, rs2069779), and *IL6* (rs2069827, rs2069837, rs2066992, rs2069840, rs2069861, rs10242595) were genotyped. For a summary of the regulatory function of the selected SNPs, see Supplementary Table 1.

Genomic DNA was extracted from blood, saliva, or buccal swab samples and DNA was extracted using the high‐salt method (Lahiri and Nurnberger, 1991). Genotyping was done blind to case status using the Taqman® OpenArray® Genotyping system (Applied Biosystems Inc, Foster City, CA) according to manufacturer's protocols.

#### 2.2.2 Publicly available genome‐wide association study (GWAS) and phenome‐wide association study (PheWAS) data

To further investigate interleukin SNPs of interest (i.e., those that were associated with internalizing behaviors in our discovery sample), we evaluated their association with internalizing behaviors in previously published publicly available GWASs of adult depression (Wray et al., 2018) and adult anxiety (Otowa et al., 2016). These GWASs have been described in detail elsewhere (Otowa et al., 2016). Briefly, the samples comprising these GWASs included a total of 135,458 adult cases and 344,901 controls for depression (Wray et al., 2018) and 17,016 adult cases and 14,745 controls for anxiety (Otowa et al., 2016).

### 2.3 Power analysis

Power calculations for main effects were done post‐hoc using Quanto version 1.2.4, assuming an additive model and MAF = 0.23 (mean MAF of SNPs included in our study). Assuming a population prevalence of 10% for childhood‐onset aggression (cases were defined as children scoring above the 90th percentile on the CBCL), we had over 80% power to detect SNPs with an OR of 1.39 in association with aggression in the sample of 212 cases and 213 controls. Assuming a population mean CBCL internalizing problems score of 59.93 ± 13.7, we had over 80% power to detect a difference in mean internalizing problems of 3.28 between genotypes in the total sample of 425 subjects. Assuming a population mean CDI total score of 49.12 ± 12.1, we had over 80% power to detect a difference in mean CDI of 2.91 between genotypes in the total sample of 425 subjects. Assuming a population mean RCMAS total score of 47.82 ± 12.6, we had over 80% power to detect a difference in mean RCMAS of 3.02 between genotypes in the total sample of 425 subjects.

### 2.4 Statistical analysis

Linkage‐disequilibrium (LD) structure, including the proportion of common variation in each of the interleukin genes, was determined using Haploview version 4.2 (Barrett et al., 2005). Descriptive analysis and data visualization were done using R version 3.1 statistical software (R Core Team, 2012) with the *rms* package (Harrell, 2014). After evaluating differences in demographic and clinical variables between cases and controls (Table 1), association analyses were done for each of the 14 SNPs individually using PLINK version 1.90b6.7v (Chang et al., 2015). Gene–environment interaction analyses were done for each of the 14 SNPs individually using R version 3.1 statistical software (R Core Team, 2012) with the *rms* package (Harrell, 2014).

We used logistic regression to test for association with aggression as a binary trait and linear regression to test for association with internalizing problems, anxiety, and depression as quantitative traits. We adjusted for age and sex in all analyses a priori, as these variables have previously been associated with aggression and internalizing behaviors (Martel, 2013). Given the non‐normal distribution of quantitative variables (i.e., CBCL internalizing score, RCMAS total scores, CDI total scores) in the total sample, approximately normal distribution of these variables within cases and controls, and significant differences in these variables between cases and controls (Table 1), we analyzed quantitative variables in cases and controls separately and then conducted inverse variance‐weighted fixed effects meta‐analysis using METAL (Willer et al., 2010). Finally, we evaluated gene–environment interactions between each of the interleukin SNPs and traumatic life experiences. For these gene–environment interaction analyses, we looked only within cases. The significance of gene–environment interactions was tested using linear regression for CBCL internalizing score, CDI total scores, and RCMAS total scores using a multiplicative model with adjustment for the main effects of each SNP and trauma history with age and sex as covariates.

To correct for multiple testing, we determined that the interleukin SNPs analyzed were equivalent to 13 independent tests using the single‐nucleotide polymorphism spectral decomposition method (Nyholt, 2004). We tested these 13 SNPs for association with four phenotypes, and also for gene–environment interactions with childhood adversity. We therefore used a significance threshold of α < 7.69×10^−4^ (0.05/13 genotypes * 5 outcomes). This significance threshold is conservative, as the overarching phenotype being investigated was internalizing behavior and three of the outcome measures used to study this phenotype were significantly correlated (Internalizing‐CDI Kendall's *r* = 0.33; *p* = 4.55×10^−24^, Internalizing‐RCMAS Kendall's *r* = 0.28; *p* = 2.42×10^−18^, CDI‐RCMAS Kendall's *r* = 0.49; *p* = 1.91×10^−52^).

## 3 RESULTS

### 3.1 Genetic architecture of *IL1B*, *IL2*, and *IL6* variants analyzed

We excluded one SNP (rs3136558) with poor clustering in allelic discrimination plots. Among the remaining 15 SNPs, call rates were > 85%. Duplicate samples (10%) were used to check genotyping accuracy, and > 99% concordance was observed. Two SNPs violated Hardy–Weinberg equilibrium and were removed from further analysis (rs13032029, *p* = 1×10^−4^ and rs2069779, *p* = 1×10^−56^). The remaining 13 SNPs were in Hardy–Weinberg equilibrium (*p* > 0.01). The 13 SNPs available for analysis captured 83% of common alleles (MAF > 0.05) in *IL1B*, 60% of common alleles in *IL2*, and 24% of common alleles in *IL6*.

### 3.2 Sample characteristics

Genetic data was available for 255 cases and 226 controls of European ancestry. Overall, 58% of the sample was male. As a group, cases were older than controls, were more likely to have a history of trauma, and had lower IQ as well as higher symptom scores. Demographic and clinical details of the sample are provided in Table 1.

We excluded 56 individuals, 43 cases, and 13 controls, with > 20% missing genotype rate (i.e., missing genotypes for more than 3 of the 13 interleukin SNPs). After quality control, 212 cases and 213 controls of European ancestry were available for analysis, resulting in a total sample of 425 individuals. The number of individuals included in each analysis varied depending on the missing data rate of the phenotypes analyzed.

### 3.3 Association of interleukin SNPs with childhood‐onset aggression

We investigated the association of each of the 13 interleukin SNPs with childhood‐onset aggression in our sample of 212 cases and 213 controls. Given that age and gender have been previously associated with aggression (Martel, 2013), we included these as covariates in all association tests. No association was observed between any of the interleukin SNPs and childhood‐onset aggression in our sample (all *p* > 7.69×10^−4^, Table 2).

**TABLE 2:** Association of interleukin SNPs with childhood‐onset aggression

Gene	SNP (chr:bp^a^)	Genotype^b^	MAF	OR^c^	p
*IL1B*	rs4849127 (chr2:112844982)	A/G	0.05	0.79	0.43
	rs16944 (chr2:112837290)	A/G	0.33	1.06	0.69
	rs1143634 (chr2:112832813)	A/G	0.22	0.98	0.92
	rs1143643 (chr2:112830725)	T/C	0.36	1.18	0.24
*IL2*	rs2069762 (chr4:122456825)	C/A	0.27	1.34	0.05
	rs2069778 (chr4:122454980)	A/G	0.15	0.87	0.48
	rs2069772 (chr4:122451978)	C/T	0.27	0.86	0.33
*IL6*	rs2069827 (chr7:22725837)	T/G	0.08	0.69	0.16
	rs2069837 (chr7:22728408)	G/A	0.07	1.29	0.37
	rs2066992 (chr7:22728630)	T/G	0.07	0.78	0.37
	rs2069840 (chr7:22728953)	G/C	0.33	0.93	0.64
	rs2069861 (chr7:22732035)	T/C	0.12	0.98	0.93
	rs10242595 (chr7:22734612)	A/G	0.31	1.16	0.32

^a^ Locations based on Genome Reference Consortium Build 37, hg19.

^b^ Genotypes are reported as A1/A2 correspond to Minor/Major alleles.

^c^ Effect sizes are reported with respect to the minor allele (A1) as the risk allele in all analyses.

### 3.4 Association of interleukin SNPs with internalizing behaviors in children

Next, we evaluated the association of each interleukin SNP with continuous measures of childhood internalizing problems (CBCL subscale), depressive symptoms (CDI total score), and anxiety symptoms (RCMAS total score). We performed association testing separately in cases and controls, and then combined the results using inverse variance‐weighted fixed effects meta‐analysis. Similar to our case–control analysis, we adjusted for age and gender in all analyses of internalizing behaviors.

We observed significant association of *IL6* variant rs2069827 with depressive symptoms (effect size among cases, *β*_CAS_ = −4.98 for each copy of the T allele; effect size among controls, *β*_CON_ = −2.80; inverse variance‐weighted fixed effects meta‐analysis of effect in cases and controls, p‐value_META_ = 7.15×10^−4^), and a trend for association of rs2069827 with anxiety (*β*_CAS_ = −3.66 for each copy of the T allele; *β*_CON_ = 0.07; p_META_ = 0.006). No other interleukin SNPs showed association with any of the internalizing behaviors investigated (all remaining p > 7.69×10^−4^, Table 3).

**TABLE 3:** Association of interleukin SNPs with internalizing behaviors

		Internalizing problems					Anxiety					Depression				
		*Cases*		*Controls*		*Combined*	*Cases*		*Controls*		*Combined*	*Cases*		*Controls*		*Combined*
Gene	SNP	*𝛽_CAS_*	*p_CAS_*	*𝛽_CON_*	*p_CON_*	*p_META_*	*𝛽_CAS_*	*p_CAS_*	*𝛽_CON_*	*p_CON_*	*p_META_*	*𝛽_CAS_*	p_CAS_	𝛽_CON_	p_CON_	p_META_
*IL1B*	rs4849127	0.54	0.78	1.05	0.65	0.61	–0.51	0.81	4.24	0.06	0.90	–3.85	0.11	3.18	0.02	0.56
	rs16944	–0.19	0.84	0.60	0.54	0.77	–1.46	0.22	1.07	0.28	0.94	–0.45	0.71	0.61	0.31	0.64
	rs1143634	–0.11	0.92	0.16	0.89	0.98	0.44	0.75	1.85	0.11	0.17	–1.74	0.22	0.39	0.58	0.65
	rs1143643	0.36	0.70	–0.45	0.66	0.98	–0.17	0.88	–1.00	0.33	0.42	1.49	0.22	–0.76	0.22	0.98
*IL2*	rs2069762	–0.85	0.40	1.33	0.24	0.83	0.05	0.97	1.14	0.31	0.45	0.002	0.99	0.85	0.23	0.39
	rs2069778	–0.52	0.69	–2.23	0.12	0.17	0.88	0.60	–0.68	0.63	0.99	1.17	0.49	–0.57	0.51	0.99
	rs2069772	2.56	0.01	–0.90	0.45	0.21	0.32	0.81	–0.09	0.94	0.91	0.80	0.54	–0.36	0.62	0.94
*IL6*	rs2069827	1.01	0.53	–0.63	0.75	0.82	–3.66	0.07	–4.09	0.04	0.006	**‐4.98**	**0.01**	**‐2.80**	**0.02**	**7.15×10^−4^**
	rs2069837	–0.07	0.97	–0.57	0.76	0.81	–0.56	0.82	0.60	0.75	0.94	2.70	0.34	0.35	0.76	0.38
	rs2066992	1.24	0.49	4.19	0.04	0.05	1.77	0.43	4.28	0.03	0.04	2.98	0.16	2.40	0.05	0.02
	rs2069840	0.49	0.61	–0.72	0.52	0.93	1.35	0.26	–0.56	0.61	0.67	1.11	0.35	0.80	0.24	0.13
	rs2069861	–1.43	0.29	–0.19	0.90	0.40	–0.66	0.71	1.54	0.32	0.65	–2.59	0.14	0.26	0.79	0.40
	rs10242595	0.38	0.70	1.85	0.08	0.13	1.52	0.22	0.67	0.53	0.19	2.09	0.10	0.17	0.79	0.18

Association between SNPs and symptom severity was tested by linear regression with adjustment for age and gender as covariates, using an additive genotypic model; *p*‐values shown are not corrected for multiple testing, p_cor_ = 13*p‐value; 𝛽_CAS_, effect size in cases; 𝛽_CON_, effect size in controls; p_CAS_, *p*‐value in cases; *p*_CON_, *p*‐value in controls; p_META_, *p*‐value in inverse variance‐weighted fixed effects meta‐analysis of effect in cases and controls.

To further explore the association of *IL6* variant rs2069827 with depression and anxiety, we evaluated the main effect of this SNP in independent GWASs of these traits (Otowa et al., 2016; Wray et al., 2018). Notably, the GWASs were of adults meeting clinical criteria for major depressive disorder (MDD) and anxiety disorders. Thus, these GWASs were not true replication samples as they comprised a different population than our discovery sample of children who did not meet clinical criteria for MDD or anxiety disorders, but were experiencing a variable number of depressive and anxious symptoms. There was no significant association of rs2069827 with MDD (OR = 1.01; *p* = 0.22) or anxiety (OR = 1.09; *p* = 0.56) in these samples, and the direction of effect was the opposite of that observed in our discovery sample with the T allele more common among cases than controls.

### 3.5 Interaction between interleukin SNPs and childhood adversity

In our sample of early life aggression cases, severe childhood adversity (MAYSI‐2 Traumatic Experiences scale score ≥4) was associated with greater severity of depressive symptoms (*β* = 10.35, p = 4.38×10^−6^) but not severity of internalizing problems (*p* = 0.08) or anxiety (*p* = 0.07) when adjusting for age and gender as covariates.

**FIGURE 1:**
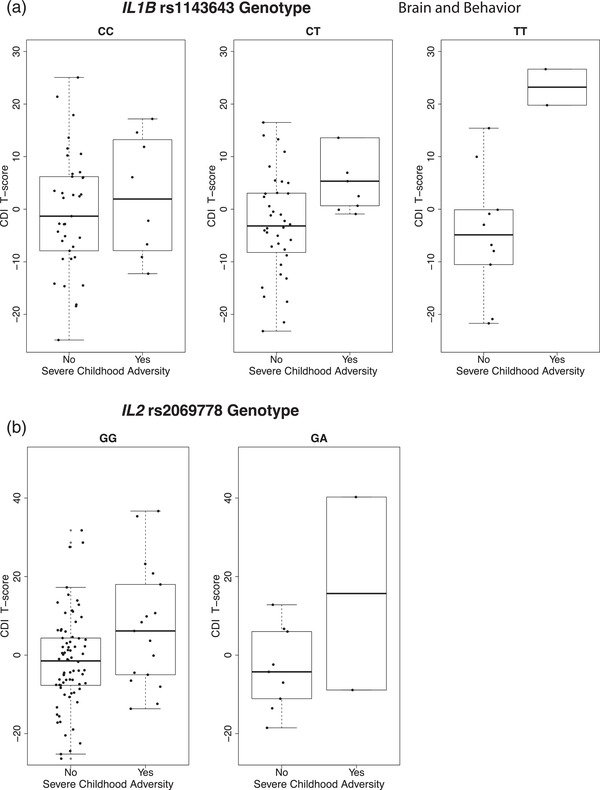
**Statistical interaction between severe childhood adversity and (a) *IL1B* rs1143643 and (b) *IL2* rs2069778 for depressive symptoms**. Depressive symptom severity (measured using the Children's Depression Inventory, CDI) was plotted stratified by exposure to severe childhood adversity (defined as MAYSI‐2 Traumatic Experiences scale score ≥4) for each genotype at (a) *IL1B* rs1143643 and (b) *IL2* rs2069778. CDI T‐scores were plotted after adjustment for age and gender as covariates using linear regression model residuals. The AA genotype for *IL2* rs2069778 is not included in (b), as only one individual in our sample had the AA genotype; this individual did not have a history of severe childhood adversity

To evaluate whether *IL1B*, *IL2*, or *IL6* genotype moderated the influence of childhood adversity on severity of depressive symptoms, we undertook exploratory gene–environment interaction analyses within cases. These analyses also adjusted for age and gender as covariates. Interactions with severe childhood adversity did not survive multiple testing correction for any of the interleukin SNPs (all *p* > 7.69×10^−4^, Table 4). We observed suggestive evidence of interaction effects between severe childhood adversity and *IL1B* variant rs1143643 and *IL2* variant rs2069778 (*p* < 0.01, Figure 1). Notably, these interaction effects were driven by a small number of individuals carrying the minor alleles for these variants in our sample (*n* < 10, Figure 1).

**TABLE 4:** Gene–environment interactions between interleukin SNPs and traumatic life experiences

Gene	SNP	𝛽	*p*
*IL1B*	rs4849127	9.56	0.36
	rs16944	–4.65	0.32
	rs1143634	–1.30	0.82
	rs1143643	13.94	4.62×10^−3^
*IL2*	rs2069762	–0.80	0.88
	rs2069778	18.24	9.23×10^−3^
	rs2069772	4.95	0.47
*IL6*	rs2069827	–13.3	0.07
	rs2069837	–22.96	0.11
	rs2066992	14.73	0.06
	rs2069840	–7.80	0.16
	rs2069861	–1.55	0.79
	rs10242595	–0.03	0.99

*Note*: Statistical interaction between each SNP and severe childhood adversity for depressive symptoms was tested by linear regression using a SNP*adversity interaction term with adjustment for main effects of SNP genotype and adversity as well as covariates age and gender; *p*‐values shown are not corrected for multiple testing, *p*_cor_ = 13*p‐value.

## 4 DISCUSSION

In this study, we provide an initial exploration of common cytokine gene variants in childhood‐onset aggression and internalizing behaviors in a deeply phenotyped Canadian sample. We performed a hypothesis‐driven study to evaluate the association of *IL1B*, *IL2*, and *IL6* gene variants with aggression and internalizing behaviors in children and adolescents, and to explore gene–environment interactions with childhood adversity.

Our findings provide preliminary evidence that *IL6* variant rs2069827 may be associated with severity of depressive symptoms in children and adolescents. Additional studies of rs2069827 in independent cohorts capturing early life depressive symptoms are needed to validate whether or not this association is robust. Notably, rs2069827 has not been previously associated with any human trait in published genome‐wide or phenome‐wide association studies (Buniello et al., 2019; Denny et al., 2013). However, rs2069827 was previously identified as nominally significant in a Dutch prospective cohort study of longevity investigating 102 SNPs in 16 genes; in this study, the T allele (which had a protective effect on depressive symptoms in our sample) was associated with increased longevity (Soerensen et al., 2013). Although rs2069827 is located in an *IL6* intron and within a putative transcription factor binding site, rs2069827 was not associated with differences in *IL6* expression in RNA expression studies (Soerensen et al., 2013) and was not identified as an expression quantitative trait locus (eQTL) for *IL6* in the Genotype‐Tissue Expression (GTEx) project (GTEx Consortium, 2017). Thus, if the preliminary association we observed between rs2069827 and early life depressive symptoms is replicated in future studies, the biological mechanism by which this SNP influences depressive symptoms may not be via altered *IL6* expression.

We also demonstrate the potential contribution of applying hypothesis‐driven gene–environment interaction analyses to understand childhood‐onset behavior problems. While we did not observe significant interactions between interleukin SNPs and childhood adversity in moderating depressive symptoms, we did observe nominally significant interaction effects for rs1143643 (*IL1B*) and rs2069778 (*IL2*). These SNPs have not been identified as associated with any human trait in published genome‐wide or phenome‐wide association studies (Buniello et al., 2019; Denny et al., 2013). However, a gene‐based study of three SNPs in *IL1B* reported that the C allele of rs1143643 was associated with non‐response to antidepressants and reduced amygdala reactivity among 256 patients with depression (Baune et al., 2010). Interestingly, two gene–environment interaction studies have reported that *IL1B* variants interact with stress to modulate depressive symptoms. In a Hungarian sample of 1,053 adult participants, rs1143643 was found to interact with recent life stress to modulate depression and anxiety (Kovacs et al., 2016). More specifically, T allele carriers were protected from depressive and anxiety symptoms when exposed to negative life events (Kovacs et al., 2016); this is the opposite effect observed in our sample of children, where T allele carriers showed greater depressive symptom severity when exposed to severe childhood adversity. In an American sample of 198 preschoolers exposed to maltreatment, the T allele of rs1143633 (which is in linkage disequilibrium with the T allele of rs1143643, *r* ^2^ = 0.98 (1000 Genomes Project Consortium, 2015)) was associated with greater depressive symptoms among children experiencing recent life stress, although no such gene–environment interaction was observed between rs1143643 and maltreatment (Ridout et al., 2014). The differences in direction of effect across studies may be accounted for by differences in study population (ancestry, age, clinical presentation) and in environmental and outcome measures. Overall, several previous studies have implicated rs1143643 in depression and in gene–environment interactions in depression, suggesting this variant is a promising target for future gene–environment interaction studies in larger childhood depression samples.

Our study was subject to several limitations. First, early life aggression and internalizing behaviors are complex traits involving hundreds of variants, and our hypothesis‐driven study did not consider the potential genetic effects of all interleukin gene variants or other genes outside of the interleukins. Second, the SNPs surviving quality control did not capture all common variations in the interleukin genes studied. Third, although we restricted our analyses to European ancestry individuals, we cannot exclude the possibility of residual population stratification given that ancestry was determined by self‐report. This is particularly notable given that rs2069827, the SNP we identified as associated with depressive symptoms in our sample, is polymorphic in European but not Asian or African populations (1000 Genomes Project Conso, 2015). Fourth, given that our sample comprised European ancestry subjects, our findings may not be generalizable to other ancestral populations. Fifth, questionnaire data capturing the outcome variables being studied were incomplete in our sample, resulting in loss of subjects available for analysis and reducing statistical power (missingness rates varied from 2% for parent‐reported problem behaviors captured using the CBCL to 5% for self‐reported depressive symptoms captured using the CDI). Finally, as we did not have a suitable replication sample, our study is exploratory in nature and requires validation in independent samples.

In summary, we investigated genetic variation in *IL1B*, *IL2*, and *IL6* as potential risk factors for early life aggression and internalizing behaviors, and explored interactions between these genes and severe childhood adversity in moderating depressive symptoms. Our findings suggest rs2069827 (*IL6*) as a potential risk variant for depressive symptoms in children and adolescents, which should be prioritized for investigation in future studies of childhood mood symptoms. We also observed nominally significant interactions between rs1143643 (*IL1B*) and rs2069778 (*IL2*) and childhood adversity in moderating depressive symptoms, whereby children and adolescents carrying minor alleles for these variants were more sensitive to the depressogenic effects of childhood adversity. While our findings are from exploratory investigations, they may be helpful in revealing insights into the role of interleukins in early life aggression and internalizing behaviors. As larger child and adolescent depression samples become available in the future through international collaboration, it will be important to examine whether our findings are replicated in statistically robust, hypothesis‐free GWAS and gene–environment analyses.

## CONFLICT OF INTEREST

James L. Kennedy is a member of the Scientific Advisory Board of Myriad Neuroscience (unpaid). James L. Kennedy and Clement C. Zai are authors on several patents relating to pharmacogenetic tests for psychiatric medications. The remaining authors have no conflicts of interest to declare.

### PEER REVIEW

The peer review history for this article is available at https://publons.com/publon/10.1002/brb3.2753

## Supporting information

Supplementary Table 1. Evidence of regulatory function for interleukin SNPs selected for genotyping

## Data Availability

Genetic data used in this study are not available to be shared due to privacy or ethical restrictions.

## References

[brb32753-bib-0001] Achenbach, T. M. , Edelbrock, C. , & Howell, C. T. (1987). Empirically based assessment of the behavioral/emotional problems of 2‐ and 3‐ year‐old children. Journal of Abnormal Child Psychology, 15(4), 629–650. 3437096 10.1007/BF00917246

[brb32753-bib-0002] Achenbach, T. M. , Howell, C. T. , Mcconaughy, S. H. , & Stanger, C. (1995). Six‐year predictors of problems in a national sample: III. Transitions to young adult syndromes. Journal of the American Academy of Child and Adolescent Psychiatry, 34(5), 658–669. 7775361 10.1097/00004583-199505000-00018

[brb32753-bib-0003] Achenbach, T. M. , & Rescorla, L. A. (2001). Manual for the ASEBA school‐age forms & profiles. Burlington, VT: Research Center for Children, Youth, & Families .

[brb32753-bib-0004] International HapMap 3 Consortium . (2010). Integrating common and rare genetic variation in diverse human populations. Nature, 467(7311), 52–58. 20811451 10.1038/nature09298 PMC3173859

[brb32753-bib-0005] Anda, R. F. , Felitti, V. J. , Bremner, J. D. , Walker, J. D. , Whitfield, C. h. , Perry, B. D. , Dube, S. h. R. , & Giles, W. H. (2006). The enduring effects of abuse and related adverse experiences in childhood. European Archives of Psychiatry and Clinical Neuroscience, 256(3), 174–186. 16311898 10.1007/s00406-005-0624-4 PMC3232061

[brb32753-bib-0006] 1000 Genomes Project Consortium . (2015). A global reference for human genetic variation. Nature, 526(7571), 68–74. 26432245 10.1038/nature15393 PMC4750478

[brb32753-bib-0007] Barrett, J. C. , Fry, B. , Maller, J. , & Daly, M. J. (2005). Haploview: Analysis and visualization of LD and haplotype maps. Bioinformatics, 21(2), 263–265. 15297300 10.1093/bioinformatics/bth457

[brb32753-bib-0008] Baune, B. T. , Dannlowski, U. , Domschke, K. , Janssen, D. G. A. , Jordan, M. A. , Ohrmann, P. , Bauer, J. , Biros, E. , Arolt, V. , Kugel, H. , Baxter, A. G. , & Suslow, T. (2010). The interleukin 1 beta (IL1B) gene is associated with failure to achieve remission and impaired emotion processing in major depression. Biological Psychiatry, 67(6), 543–549. 20044070 10.1016/j.biopsych.2009.11.004

[brb32753-bib-0009] Beitchman, J. H. , Baldassarra, L. , Mik, H. , De Luca, V. , King, N. , Bender, D. , Ehtesham, S. , & Kennedy, J. L. (2006). Serotonin transporter polymorphisms and persistent, pervasive childhood aggression. American Journal of Psychiatry, 163(6), 1103–1105. 16741214 10.1176/ajp.2006.163.6.1103

[brb32753-bib-0010] Beitchman, J. H. , Mik, H. M. , Ehtesham, S. , Douglas, L. , & Kennedy, J. L. (2004). MAOA and persistent, pervasive childhood aggression. Molecular Psychiatry, 9(6), 546–547. 15024395 10.1038/sj.mp.4001492

[brb32753-bib-0011] Belem da Silva, C. T. , de Abreu Costa, M. , Kapczinski, F. , Wollenhaupt de Aguiar, B. , Salum, G. A. , & Manfro, G. G. (2017). Inflammation and internalizing disorders in adolescents. Progress in Neuro‐Psychopharmacology & Biological Psychiatry, *77*, 7133–7137. 10.1016/j.pnpbp.2017.03.023 28385493

[brb32753-bib-0012] Benke, K. S. , Nivard, M. G. , Velders, F. P. , Walters, R. K. , Pappa, I. , Scheet, P. A. , Xiao, X. , Ehli, E. A. , Palmer, L. J. , Whitehouse, A. J. O. , Verhulst, F. C. , Jaddoe, V. W. , Rivadeneira, F. , Groen‐Blokhuis, M. M. , Van Beijsterveldt, C. E. M. , Davies, G. E. , Hudziak, J. J. , Lubke, G. H. , Boomsma, D. I. , … Middeldorp, C. M. (2014). A genome‐wide association meta‐analysis of preschool internalizing problems. Journal of the American Academy of Child and Adolescent Psychiatry, 53(6), 667–676. e7. 24839885 10.1016/j.jaac.2013.12.028

[brb32753-bib-0013] Bergen, S. E. , Gardner, C. O. , & Kendler, K. S. (2007). Age‐related changes in heritability of behavioral phenotypes over adolescence and young adulthood: A meta‐analysis. Twin Res Hum Genet, 10(3), 423–433. 17564500 10.1375/twin.10.3.423

[brb32753-bib-0014] Bhatt, S. , Zalcman, S. , Hassanain, M. , & Siegel, A. (2005). Cytokine modulation of defensive rage behavior in the cat: Role of GABAA and interleukin‐2 receptors in the medial hypothalamus. Neuroscience, 133(1), 17–28. 15893628 10.1016/j.neuroscience.2005.01.065

[brb32753-bib-0015] Boyle, A. P. , Hong, E. L. , Hariharan, M. , Cheng, Y. , Schaub, M. A. , Kasowski, M. , Karczewski, K. J. , Park, J. , Hitz, B. C. , Weng, S. , Cherry, J. M. , & Snyder, M. (2012). Annotation of functional variation in personal genomes using RegulomeDB. Genome Research, 22(9), 1790–1797. 22955989 10.1101/gr.137323.112 PMC3431494

[brb32753-bib-0016] Buniello, A. , Macarthur, J. A. L. , Cerezo, M. , Harris, L. W. , Hayhurst, J. , Malangone, C. , Mcmahon, A. , Morales, J. , Mountjoy, E. , Sollis, E. , Suveges, D. , Vrousgou, O. , Whetzel, P. L. , Amode, R. , Guillen, J. A. , Riat, H. S. , Trevanion, S. J. , Hall, P. , …, & Parkinson, H. (2019). The NHGRI‐EBI GWAS Catalog of published genome‐wide association studies, targeted arrays and summary statistics 2019. Nucleic Acids Research, 47(D1), D1005–D1012. 30445434 10.1093/nar/gky1120 PMC6323933

[brb32753-bib-0017] Campbell, S. B. (1995). Behavior problems in preschool children: A review of recent research. Journal of Child Psychology and Psychiatry and Allied Disciplines, 36(1), 113–149. 7714027 10.1111/j.1469-7610.1995.tb01657.x

[brb32753-bib-0018] Campbell, S. B. , & Ewing, L. J. (1990). Follow‐up of hard‐to‐manage preschoolers: Adjustment at age 9 and predictors of continuing symptoms. Journal of Child Psychology and Psychiatry and Allied Disciplines, 31(6), 871–889. 2246339 10.1111/j.1469-7610.1990.tb00831.x

[brb32753-bib-0019] Caspi, A. , Mcclay, J. , Moffitt, T. E. , Mill, J. , Martin, J. , Craig, I. W. , Taylor, A. , & Poulton, R. (2002). Role of genotype in the cycle of violence in maltreated children. Science, 297(5582), 851–854. 12161658 10.1126/science.1072290

[brb32753-bib-0020] Chang, C. C. , Chow, C. C. , Tellier, L. C. , Vattikuti, S. , Purcell, S. M. , & Lee, J. J. (2015). Second‐generation PLINK: Rising to the challenge of larger and richer datasets. Gigascience, *4*, 7. 25722852 10.1186/s13742-015-0047-8 PMC4342193

[brb32753-bib-0021] Chourbaji, S. , Urani, A. , Inta, I. , Sanchis‐Segura, C. , Brandwein, C. , Zink, M. , Schwaninger, M. , & Gass, P. (2006). IL‐6 knockout mice exhibit resistance to stress‐induced development of depression‐like behaviors. Neurobiology of Disease, 23(3), 587–594. 16843000 10.1016/j.nbd.2006.05.001

[brb32753-bib-0022] Cole, S. W. , Conti, G. , Arevalo, J. M. G. , Ruggiero, A. M. , Heckman, J. J. , & Suomi, S. J. (2012). Transcriptional modulation of the developing immune system by early life social adversity. The Proceedings of the National Academy of Sciences, 109(50), 20578–20583. 10.1073/pnas.1218253109 PMC3528538 23184974

[brb32753-bib-0023] Copeland, W. E. , Shanahan, L. , Worthman, C. , Angold, A. , & Costello, E. J. (2012). Generalized anxiety and C‐reactive protein levels: A prospective, longitudinal analysis. Psychological Medicine, 42(12), 2641–2650. 22716910 10.1017/S0033291712000554 PMC3449031

[brb32753-bib-0024] Costello, H. , Gould, R. L. , Abrol, E. , & Howard, R. (2019). Systematic review and meta‐analysis of the association between peripheral inflammatory cytokines and generalised anxiety disorder. BMJ Open, 9(7), e027925. 10.1136/bmjopen-2018-027925 PMC6661660 31326932

[brb32753-bib-0025] Denny, J. C. , Bastarache, L. , Ritchie, M. D. , Carroll, R. J. , Zink, R. , Mosley, J. D. , Field, J. R. , Pulley, J. M. , Ramirez, A. H. , Bowton, E. , Basford, M. A. , Carrell, D. S. , Peissig, P. L. , Kho, A. N. , Pacheco, J. A. , Rasmussen, L. V. , Crosslin, D. R. , Crane, P. K. , Pathak, J. , …, Roden, D. M. (2013). Systematic comparison of phenome‐wide association study of electronic medical record data and genome‐wide association study data. Nature Biotechnology, 31(12), 1102–1110. 10.1038/nbt.2749 PMC3969265 24270849

[brb32753-bib-0026] Dick, D. M. , Agrawal, A. , Keller, M. C. , Adkins, A. , Aliev, F. , Monroe, S. , Hewitt, J. K. , Kendler, K. S. , & Sher, K. J. (2015). Candidate gene–environment interaction research: Reflections and recommendations. Perspectives on Psychological Science, 10(1), 37–59. 25620996 10.1177/1745691614556682 PMC4302784

[brb32753-bib-0027] Dodge, K. A. , Bates, J. E. , & Pettit, G. S. (1990). Mechanisms in the cycle of violence. Science, 250(4988), 1678–1683. 2270481 10.1126/science.2270481

[brb32753-bib-0028] The ENCODE Project Consortium . (2012). An integrated encyclopedia of DNA elements in the human genome. Nature, 489(7414), 57–74. 22955616 10.1038/nature11247 PMC3439153

[brb32753-bib-0029] Graham, J. E. , Robles, T. F. , Kiecolt‐Glaser, J. K. , Malarkey, W. B. , Bissell, M. G. , & Glaser, R. (2006). Hostility and pain are related to inflammation in older adults. Brain, Behavior, and Immunity, 20(4), 389–400. 16376518 10.1016/j.bbi.2005.11.002

[brb32753-bib-0030] Grisso, T. , & Barnum, R. (2014). Massachusetts Youth Screening Instrument‐2 (MAYSI‐2): User's manual and technical report. Sarasota, FL: Professional Resource Press. setts.

[brb32753-bib-0031] GTEx Consortium . (2017). Genetic effects on gene expression across human tissues. Nature, 550(7675), 204–213. 29022597 10.1038/nature24277 PMC5776756

[brb32753-bib-0032] Harrell, F. E. J. (2014). rms: Regression Modeling Strategies. R package version 4.2‐0. http://CRAN.R‐project.org/package=rms

[brb32753-bib-0033] Hassanain, M. , Zalcman, S. , Bhatt, S. , & Siegel, A. (2003). Interleukin‐1beta in the hypothalamus potentiates feline defensive rage: Role of serotonin‐2 receptors. Neuroscience, 120(1), 227–233. 12849755 10.1016/s0306-4522(03)00264-1

[brb32753-bib-0034] Hudziak, J. J. , Van Beijsterveldt, C. E. M. , Bartels, M. , Rietveld, M. J. H. , Rettew, D. C. , Derks, E. M. , & Boomsma, D. I. (2003). Individual differences in aggression: Genetic analyses by age, gender, and informant in 3‐, 7‐, and 10‐year‐old Dutch twins. Behavior Genetics, 33(5), 575–589. 14574134 10.1023/a:1025782918793

[brb32753-bib-0035] Hughes, A. A. , Lourea‐Waddell, B. , & Kendall, P. C. (2008). Somatic complaints in children with anxiety disorders and their unique prediction of poorer academic performance. Child Psychiatry and Human Development, 39(2), 211–220. 17786552 10.1007/s10578-007-0082-5

[brb32753-bib-0036] Jami, E. S. , Hammerschlag, A. R. , & Ip, H. F. (2022). Genome‐wide association meta‐analysis of childhood and adolescent internalizing symptoms. *Journal of the American Academy of Child & Adolescent , 61*(7), 934–945. 10.1016/j.jaac.2021.11.035 PMC10859168 35378236

[brb32753-bib-0037] Janusek, L. W. , Tell, D. , Gaylord‐Harden, N. , & Mathews, H. L. (2017). Relationship of childhood adversity and neighborhood violence to a proinflammatory phenotype in emerging adult African American men: An epigenetic link. Brain, Behavior, and Immunity, *60*, 126–135. 27765646 10.1016/j.bbi.2016.10.006

[brb32753-bib-0038] Johnson, D. , Dupuis, G. , Piche, J. , Clayborne, Z. , & Colman, I. (2018). Adult mental health outcomes of adolescent depression: A systematic review. Depression and Anxiety, 35(8), 700–716. 29878410 10.1002/da.22777

[brb32753-bib-0039] Koo, J. W. , & Duman, R. S. (2008). IL‐1beta is an essential mediator of the antineurogenic and anhedonic effects of stress. The Proceedings of the National Academy of Sciences, 105(2), 751–756. 10.1073/pnas.0708092105 PMC2206608 18178625

[brb32753-bib-0040] Kovacs, D. , Eszlari, N. , Petschner, P. , Pap, D. , Vas, S. , Kovacs, P. , Gonda, X. , Juhasz, G. , & Bagdy, G. (2016). Effects of IL1B single nucleotide polymorphisms on depressive and anxiety symptoms are determined by severity and type of life stress. Brain, Behavior, and Immunity, *56*, 96–104. 10.1016/j.bbi.2016.02.012 26891860

[brb32753-bib-0041] Kovacs, M. (1985). Children's Depression Inventory (CDI). *Psychopharmacology Bulletin, 21*(4), 995‐998. 4089116

[brb32753-bib-0042] Lahiri, D. K. , & Numberger, J. I. (1991). A rapid non‐enzymatic method for the preparation of HMW DNA from blood for RFLP studies. Nucleic Acids Research, 19(19), 5444. 1681511 10.1093/nar/19.19.5444 PMC328920

[brb32753-bib-0043] Lamers, F. , Milaneschi, Y. , Smit, J. H. , Schoevers, R. A. , Wittenberg, G. , & Penninx, B. W. J. H. (2019). Longitudinal association between depression and inflammatory markers: Results from the Netherlands Study of Depression and Anxiety. Biological Psychiatry, 85(10), 829–837. 30819515 10.1016/j.biopsych.2018.12.020

[brb32753-bib-0044] Locher, C. , Koechlin, H. , Zion, S. R. , Werner, C. , Pine, D. S. , Kirsch, I. , Kessler, R. C. , & Kossowsky, J. (2017). Efficacy and safety of selective serotonin reuptake inhibitors, serotonin‐norepinephrine reuptake inhibitors, and placebo for common psychiatric disorders among children and adolescents. JAMA Psychiatry, 74(10), 1011–1020. 28854296 10.1001/jamapsychiatry.2017.2432 PMC5667359

[brb32753-bib-0045] Malik, A. I. , Zai, C. C. , Berall, L. , Abu, Z. , Din, F. , Nowrouzi, B. , Chen, S. , & Beitchman, J. H. (2014). The role of genetic variants in genes regulating the oxytocin‐vasopressin neurohumoral system in childhood‐onset aggression. Psychiatric Genetics, 24(5), 201–210. 24871896 10.1097/YPG.0000000000000044

[brb32753-bib-0046] Martel, M. M. (2013). Sexual selection and sex differences in the prevalence of childhood externalizing and adolescent internalizing disorders. Psychological Bulletin, 139(6), 1221–1259. 23627633 10.1037/a0032247

[brb32753-bib-0047] Michelson, D. , Davenport, C. , Dretzke, J. , Barlow, J. , & Day, C. (2013). Do evidence‐based interventions work when tested in the “real world?” A systematic review and meta‐analysis of parent management training for the treatment of child disruptive behavior. Clinical Child and Family Psychology Review, 16(1), 18–34. 23420407 10.1007/s10567-013-0128-0

[brb32753-bib-0048] Miller, A. H. , & Raison, C. L. (2016). The role of inflammation in depression: From evolutionary imperative to modern treatment target. Nature Reviews Immunology, 16(1), 22–34. 10.1038/nri.2015.5 PMC5542678 26711676

[brb32753-bib-0049] Mitchell, R. H. B. , & Goldstein, B. I. (2014). Inflammation in children and adolescents with neuropsychiatric disorders: A systematic review. Journal of the American Academy of Child and Adolescent Psychiatry, 53(3), 274–296. 24565356 10.1016/j.jaac.2013.11.013

[brb32753-bib-0050] Moffitt, T. E. (1990). Juvenile delinquency and attention deficit disorder: Boys' developmental trajectories from age 3 to age 15. Child Development, 61(3), 893–910. 2364762 10.1111/j.1467-8624.1990.tb02830.x

[brb32753-bib-0051] Nemeroff, C. B. (2016). Paradise lost: The neurobiological and clinical consequences of child abuse and neglect. Neuron, 89(5), 892–909. 26938439 10.1016/j.neuron.2016.01.019

[brb32753-bib-0052] Niraula, A. , Witcher, K. G. , Sheridan, J. F. , & Godbout, J. P. (2019). Interleukin‐6 induced by social stress promotes a unique transcriptional signature in the monocytes that facilitate anxiety. Biological Psychiatry, 85(8), 679–689. 30447911 10.1016/j.biopsych.2018.09.030 PMC6440848

[brb32753-bib-0053] Nusslock, R. , & Miller, G. E. (2016). Early‐life adversity and physical and emotional health across the lifespan: A neuroimmune network hypothesis. Biological Psychiatry, 80(1), 23–32. 26166230 10.1016/j.biopsych.2015.05.017 PMC4670279

[brb32753-bib-0054] Otowa, T. , Hek, K. , Lee, M. , Byrne, E. M. , Mirza, S. S. , Nivard, M. G. , Bigdeli, T. , Aggen, S. H. , Adkins, D. , Wolen, A. , Fanous, A. , Keller, M. C. , Castelao, E. , Kutalik, Z. , Der Auwera, S. V. , Homuth, G. , Nauck, M. , Teumer, A. , Milaneschi, Y. , … Hettema, J. M. (2016). Meta‐analysis of genome‐wide association studies of anxiety disorders. Molecular Psychiatry, 21(10), 1391–1399. 26754954 10.1038/mp.2015.197 PMC4940340

[brb32753-bib-0055] Pappa, I. , St Pourcain, B. , Benke, K. , Cavadino, A. , Hakulinen, C. , Nivard, M. G. , Nolte, I. M. , Tiesler, C. M. T. , Bakermans‐Kranenburg, M. J. , Davies, G. E. , Evans, D. M. , Geoffroy, M. ‐ C. , Grallert, H. , Groen‐Blokhuis, M. M. , Hudziak, J. J. , Kemp, J. P. , Keltikangas‐Järvinen, L. , Mcmahon, G. , Mileva‐Seitz, V. R. , … Tiemeier, H. (2016). A genome‐wide approach to children's aggressive behavior: The EAGLE consortium. American Journal of Medical Genetics. Part B, Neuropsychiatric Genetics, 171(5), 562–572. 10.1002/ajmg.b.32333 26087016

[brb32753-bib-0056] Pham, C. , Bekkering, S. , O'Hely, M. , Burgner, D. , Thomson, S. , Vuillermin, P. , Collier, F. , Marx, W. , Mansell, T. , Symeonides, C. , Sly, P. D. , Tang, M. L. K. , Saffery, R. , & Ponsonby, A.‐L. (2022). Infant inflammation predicts childhood emotional and behavioral problems and partially mediates socioeconomic disadvantage. Brain, Behavior, and Immunity, *104*, 83–94. 10.1016/j.bbi.2022.05.011 35618227

[brb32753-bib-0057] Pikard, J. , Roberts, N. , & Groll, D. (2018). Pediatric referrals for urgent psychiatric consultation: Clinical characteristics, diagnoses and outcome of 4 to 12 year old children. Journal of the Canadian Academy of Child and Adolescent Psychiatry, 27(4), 245–251. 30487940 PMC6254263

[brb32753-bib-0058] Pine, D. S. , Cohen, P. , Gurley, D. , Brook, J. , & Ma, Y. (1998). The risk for early‐adulthood anxiety and depressive disorders in adolescents with anxiety and depressive disorders. Archives of General Psychiatry, 55(1), 56–64. 9435761 10.1001/archpsyc.55.1.56

[brb32753-bib-0059] Pringsheim, T. , & Gorman, D. (2012). Second‐generation antipsychotics for the treatment of disruptive behaviour disorders in children: A systematic review. Canadian Journal of Psychiatry Revue Canadienne De Psychiatrie, 57(12), 722–727. 23228230 10.1177/070674371205701203

[brb32753-bib-0060] Pringsheim, T. , Lam, D. , Ching, H. , & Patten, S. (2011). Metabolic and neurological complications of second‐generation antipsychotic use in children. Drug Safety, 34(8), 651–668. 21751826 10.2165/11592020-000000000-00000

[brb32753-bib-0061] R Core Team . (2012). R: A language and environment for statistical computing. R Foundation for Statistical Computing. http://www.R‐project.org/

[brb32753-bib-0062] Reynolds, C. R. , & Richmond, B. O. (1985) . Revised Children's Manifest Anxiety Scale . RCMAS Manual. Los Angeles: Western Psychological Services.

[brb32753-bib-0063] Rhee, S. H. , & Waldman, I. D. (2002). Genetic and environmental influences on antisocial behavior: A meta‐analysis of twin and adoption studies. Psychological Bulletin, 128(3), 490–529. 12002699

[brb32753-bib-0064] Ridout, K. K. , Parade, S. H. , Seifer, R. , Price, L. H. , Gelernter, J. , Feliz, P. , & Tyrka, A. R. (2014). Interleukin 1B gene (IL1B) variation and internalizing symptoms in maltreated preschoolers. Development and Psychopathology, 26(4 Pt 2), 1277–1287. 25422961 10.1017/S0954579414001023 PMC4459595

[brb32753-bib-0065] Saarinen, A. , Keltikangas‐Järvinen, L. , Dobewall, H. , Ahola‐Olli, A. , Salmi, M. , Lehtimäki, T. , Raitakari, O. , Jalkanen, S. , & Hintsanen, M. (2021). Risky emotional family environment in childhood and depression‐related cytokines in adulthood: The protective role of compassion. Developmental Psychobiology, 63(5), 1190–1201. 33421111 10.1002/dev.22070

[brb32753-bib-0066] Slopen, N. , Kubzansky, L. D. , & Koenen, K. C. (2013). Internalizing and externalizing behaviors predict elevated inflammatory markers in childhood. Psychoneuroendocrinology, 38(12), 2854–2862. 24011503 10.1016/j.psyneuen.2013.07.012

[brb32753-bib-0067] Soerensen, M. , Dato, S. , Tan, Q. , Thinggaard, M. , Kleindorp, R. , Beekman, M. , Suchiman, H. E. D. , Jacobsen, R. , Mcgue, M. , Stevnsner, T. , Bohr, V. A. , De Craen, A. J. M. , Westendorp, R. G. J. , Schreiber, S. , Slagboom, P. E. , Nebel, A. , Vaupel, J. W. , Christensen, K. , & Christiansen, L. (2013). Evidence from case‐ control and longitudinal studies supports associations of genetic variation in APOE, CETP, and IL6 with human longevity. Age (Dordr), 35(2), 487–500. 22234866 10.1007/s11357-011-9373-7 PMC3592963

[brb32753-bib-0068] Sukhodolsky, D. G. , Solomon, R. M. , & Perine, J. (2000). Cognitive‐behavioral, anger‐control intervention for elementary school children: A treatment‐outcome study. Journal of Child and Adolescent Group Therapy, *10*, 159–170.

[brb32753-bib-0069] Takahashi, A. , Flanigan, M. E. , McEwen, B. S. , & Russo, S. J. (2018). Aggression, social stress, and the immune system in humans and animal models. Frontiers in Behavioral Neuroscience, *12*, 56. 29623033 10.3389/fnbeh.2018.00056 PMC5874490

[brb32753-bib-0070] Trzaskowski, M. , Eley, T. C. , Davis, O. S. P. , Doherty, S. J. , Hanscombe, K. B. , Meaburn, E. L. , Haworth, C. M. A. , Price, T. , & Plomin, R. (2013). First genome‐wide association study on anxiety‐related behaviours in childhood. PloS One, 8(4), e58676. 23565138 10.1371/journal.pone.0058676 PMC3614558

[brb32753-bib-0071] Tursich, M. , Neufeld, R. W. J. , Frewen, P. A. , Harricharan, S. , Kibler, J. L. , Rhind, S. G. , & Lanius, R. A. (2014). Association of trauma exposure with proinflammatory activity: A transdiagnostic meta‐analysis. Translational Psychiatry, *4*(7), e413. 10.1038/tp.2014.56 PMC4119223 25050993

[brb32753-bib-0072] Valkanova, V. , Ebmeier, K. P. , & Allan, C. L. (2013). CRP, IL‐6 and depression: A systematic review and meta‐analysis of longitudinal studies. Journal of Affective Disorders, 150(3), 736–744. 23870425 10.1016/j.jad.2013.06.004

[brb32753-bib-0073] Van Beijsterveldt, C. E. M. , Bartels, M. , Hudziak, J. J. , & Boomsma, D. I. (2003). Causes of stability of aggression from early childhood to adolescence: A longitudinal genetic analysis in Dutch twins. Behavior Genetics, 33(5), 591–605. 14574135 10.1023/a:1025735002864

[brb32753-bib-0074] Ward, L. D. , & Kellis, M. (2012). HaploReg: A resource for exploring chromatin states, conservation, and regulatory motif alterations within sets of genetically linked variants. Nucleic Acids Research, 40(Database issue), D930–D934. 22064851 10.1093/nar/gkr917 PMC3245002

[brb32753-bib-0075] Weschler, D. (1991). Wechsler Intelligence Scale for Children (3rd ed.). (WISC‐III): Manual. San Antonio, TX: The Psychological Corporation.

[brb32753-bib-0076] Willer, C. J. , Li, Y. , & Abecasis, G. R. (2010). METAL: Fast and efficient meta‐analysis of genomewide association scans. Bioinformatics, 26(17), 2190–2191. 20616382 10.1093/bioinformatics/btq340 PMC2922887

[brb32753-bib-0077] Wray, N. R. , Ripke, S. , Mattheisen, M. , Trzaskowski, M. , Byrne, E. M. , Abdellaoui, A. , Adams, M. J. , Agerbo, E. , Air, T. M. , Andlauer, T. M. F. , Bacanu, S. ‐. A. , Bækvad‐Hansen, M. , Beekman, A. F. T. , Bigdeli, T. B. , Binder, E. B. , Blackwood, D. R. H. , Bryois, J. , Buttenschøn, H. N. , Bybjerg‐Grauholm, J. , … Sullivan, P. F. (2018). Genome‐wide association analyses identify 44 risk variants and refine the genetic architecture of major depression. Nature Genetics, 50(5), 668–681. 29700475 10.1038/s41588-018-0090-3 PMC5934326

[brb32753-bib-0079] Nyholt, D. R. (2004). A simple correction for multiple testing for single‐nucleotide polymorphisms in linkage disequilibrium with each other. *The American Journal of Human Genetics*, *74*(4), 765–769. 10.1086/383251 PMC1181954 14997420

