# Supplementary material for: Exploring the association of interleukin polymorphisms with aggression and internalizing behaviors in children and adolescents
Source: Brain Behav. 2022 Sep 28;12(11):e2753. doi: 10.1002/brb3.2753 (PMC9660418; doi:10.1002/brb3.2753)
Supplement: Supplementary file 1 — Supplementary Table 1. Evidence of regulatory function for interleukin SNPs selected for genotyping [file BRB3-12-e2753-s001.docx]

**Supplementary Table 1. Evidence of regulatory function for interleukin SNPs selected for genotyping**

|  | **Chr** | **Position (bp)** | **Genic Location** | **Regulatory motifs altered^a^** | **Brain histone modifications^b^** | **Immune eQTL^c^** | **Brain eQTL^d^** | **Brain mQTL^e^** | **GWAS hits** |
| --- | --- | --- | --- | --- | --- | --- | --- | --- | --- |
| *IL1B* | 2 | 112,829,759-112,836,843 |  |  |  |  |  |  |  |
| rs4849127 |  | 112844982 | 8.1kb 5’ of *IL1B* |  |  |  |  |  |  |
| rs13032029^f^ |  | 112842838 | 6.0kb 5’ of *IL1B* | Cdp5, GATA, Pax8, Pbx1 | H3K4me1, H3K27ac | *IL1B* |  | *IL1B* |  |
| rs16944 |  | 112837290 | 447bp 5’ of *IL1B* | Maf | H3K4me1, H3K27ac, H3K4me3 | *IL1B* |  | *IL1B* |  |
| rs3136558^g^ |  | 112833698 | Intronic | Esr1, Mef2, Rar, Rora, Rxra | H3K4me1, H3K27ac, H3K4me3 |  | *AC079922.3* |  |  |
| rs1143634 |  | 112832813 | Synonymous |  | H3K4me1, H3K27ac, H3K4me3 | *SLC20A1*, *CHCHD5* | *AC079922.3* |  |  |
| rs1143643 |  | 112830725 | Intronic | Myb1 | H3K4me1 | *IL1B* |  |  |  |
| *IL2* | 4 | 122,449,480-122,456,495 |  |  |  |  |  |  |  |
| rs2069762 |  | 122456825 | 330bp 5' of IL2 | Cebpg, Hoxa10,  Ik-2, Sox13 |  |  |  |  |  |
| rs2069778 |  | 122454980 | Intronic | Pax2 |  |  |  |  |  |
| rs2069779^f^ |  | 122452742 | Intronic | Arid5a, Barx2, Dbx1, Dbx2, Dlx2, Gbx1, Hlx1, Lhx3, Ncx, Nkx, Pou, Prrx, Sox, Tef, TATA |  |  |  |  |  |
| rs2069772 |  | 122451978 | Intronic | Arid3a, Barx2, Dbx2, En1, Evi1, Gbx1, Gbx2, Hnf1, Hlxb9, Hox, Msx1, Msx2, Ncx, Nkx6, Nobox, Pax, Phox2a, Pou, Prrx1, Sox |  |  | *KIAA1109* |  | Allergic sensitization {Waage et al., 2018, #41843} |
| *IL6^h^* | 7 | 22,725,890-22,732,002 |  |  |  |  |  |  |  |
| rs2069827 |  | 22725837 | 47bp 5’ of IL6 | Cdp2 | H3Kme1, H3K27ac, H3K4me3, H3K9ac | *ADH6* | *TOMM7, KLHL7-AS1, AC005082.1* |  |  |
| rs2069837 |  | 22728408 | Intronic | Arid, Fox, Nkx62, Mef2, TATA | H3Kme1, H3K27ac, H3K4me3, H3K9ac |  |  |  | Longevity {Zeng et al., 2016, #88972}, Takayasu arteritis {Renauer et al., 2015, #80721} |
| rs2066992 |  | 22728630 | Intronic | Ctcf,  NF-kB, Rad21, Smc3, Sox2 | H3Kme1, H3K27ac, H3K4me3, H3K9ac |  |  | *IL6* |  |
| rs2069840 |  | 22728953 | Intronic | Gcnf, Rxra | H3Kme1, H3K27ac, H3K4me3 |  |  |  |  |
| rs2069861 |  | 22732035 | 32bp 3’ of IL6 | Cart1, Fox, Mef2, TATA |  |  |  |  |  |
| rs10242595 |  | 22734612 | 2.6kb 3’ of IL6 | Smc3, P300 |  |  |  | *IL6* |  |

^a^Altered regulatory motifs reported are from motif analysis of 427 human ChIP-seq datasets {Kheradpour and Kellis, 2014, #34561}; ^b^Regulatory chromatin states reported are from histone ChIP-Seq experiments {Roadmap et al., 2015, #93919}. H3K4me1 and H3K27ac histone markers correspond to enhancer regions. H3K4me3 and H3K9ac histone markers correspond to promoter regions; ^c^Immune eQTLs reported are from eQTL profiles in peripheral blood monocytes {Zeller et al., 2010, #13655}, lymphoblastoid cells {Lappalainen et al., 2013, #52039}, or whole blood {Westra et al., 2013, #5151}; ^d^Brain eQTLs reported are from a meta-analysis of summary level data from three brain eQTL studies {Qi et al., 2018, #42844}; ^e^Brain mQTLs reported are from a meta-analysis of summary level data from five mQTL studies (three measured methylation in brain, two measured methylation in peripheral blood) {Qi et al., 2018, #42844}; ^f^This SNP was not in Hardy Weinberg equilibrium, and was therefore not included in further analyses; ^g^This SNP had poor clustering in allelic discrimination plots, and was therefore not included in further analyses; ^h^IL6 has multiple splicing variants.
